# Obstructing Hamartomatous Polyp in Peutz-Jeghers Syndrome

**DOI:** 10.1155/2013/595341

**Published:** 2013-03-28

**Authors:** Brian S. Bentley, Hassan M. Hal

**Affiliations:** Department of Radiology, Penn State Milton S. Hershey Medical Center, 100 University Drive, Hershey, PA 17033, USA

## Abstract

A 53-year-old male presented with complaints of abdominal pain and weight loss. On physical exam he was noted to have mucocutaneous pigmentation around his lips and oral mucosa. Radiologic and endoscopic investigations demonstrated an obstructing mass in the second portion of the duodenum along with additional smaller soft tissue masses throughout the bowel. Histology of biopsied specimens revealed architectural disorganization without dysplasia, suggestive of Peutz-Jeghers hamartomatous polyps.

## 1. Introduction

The hamartomatous polyposis syndromes are a collection of similar diseases which have in common the tendency to develop benign polyps throughout the GI tract [1]. Peutz-Jeghers syndrome (PJS) is one of the most relatively common hamartomatous polyposis syndromes. Other polyposis syndromes include Juvenile Polyposis, PTEN hamartoma syndrome (PTEN hamartoma tumor syndrome (PHTS) is a spectrum of disorders caused by mutations of the PTEN gene), and other rarer syndromes. 

PJS was first formally described by Jeghers et al. in 1949 as a polyposis of the small intestine with melanin deposition around the mouth and lips exhibiting an autosomal dominant pattern of inheritance [2]. Although originally described as a small bowel polyposis, subsequent publications have described polyps throughout the GI tract including the stomach, small intestine, colon, and rectum [3].

PJS is a rare disease with an estimated prevalence ranging from 1 : 25,000 to 1 : 280,000 [4]. Together all of the hamartomatous polyposis syndromes account for less than one percent of all colon cancer cases in North America [5].

PJS can present in childhood with pigmentation appearing around two years of age. Cutaneous findings are classically dark blue or brown spots, 1–5 mm in size, located along the vermillion border of the lips. This pigmentation may fade with age; however, pigmentation in the buccal mucosa will typically persist and is seen in up to 95 percent of patients [3]. Pigmentation is secondary to the presence of pigmented laden macrophages in the dermis. Conversely, common freckles do not involve the buccal mucosa, are not typically seen around the lips, and are therefore readily distinguished from the mucocutaneous pigmentation seen in PJS [6]. The childhood presentation may also include multiple episodes of small bowel intussusception or obstruction which may require surgical intervention. Most patients will present by the third decade of life with a GI related complaint that will prompt further investigation and ultimately diagnosis.

## 2. Case Report

**Figure 1:**
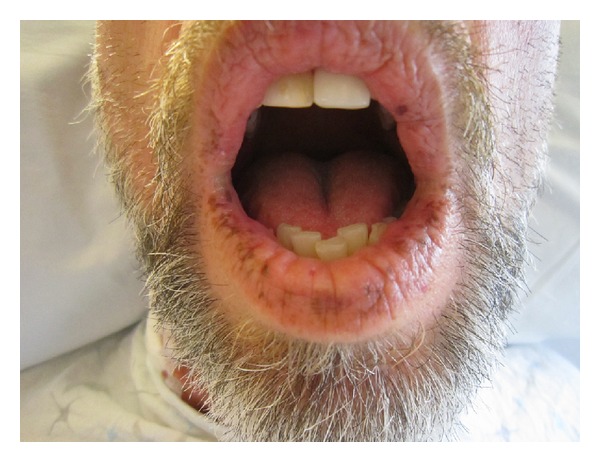
Oral mucocutaneous pigmentation characteristic of Peutz-Jeghers syndrome.

A 53-year-old male presented with complaints of peri-umbilical abdominal pain and unintentional 40 pound weight loss. The patient also reported emesis following moderate sized meals which improved when he transitioned to a liquid diet. He had a history of partial colectomy performed 25 years prior at an outside facility for a nonspecified polyposis syndrome with dysplasia. On physical exam he was noted to have mucocutaneous pigmentation around his lips and oral mucosa which went unnoticed at the time of his colectomy (Figure 1). The patient's son also has perioral pigmentation. Since his partial colectomy surgery, the patient had been on a three-year colonoscopic surveillance schedule for his remaining colon; however, his upper GI tract had never been evaluated.

**Figure 2:**
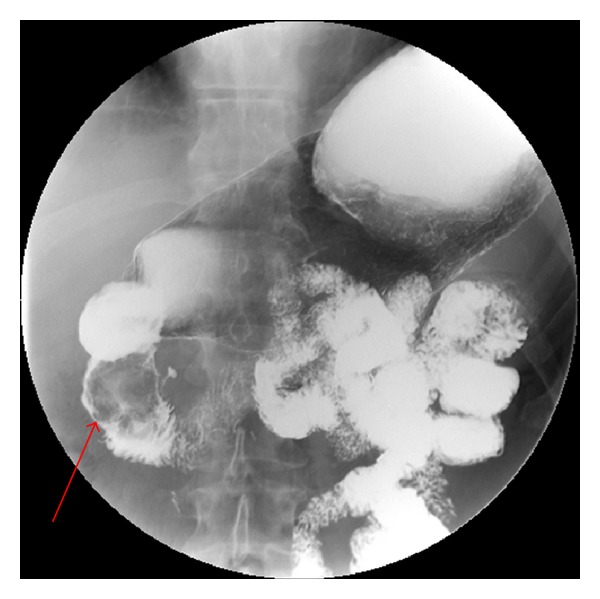
Image from upper GI series demonstrating a large filling defect (arrow) in the second portion of the duodenum (should put arrow pointing to lesion).

**Figure 3:**
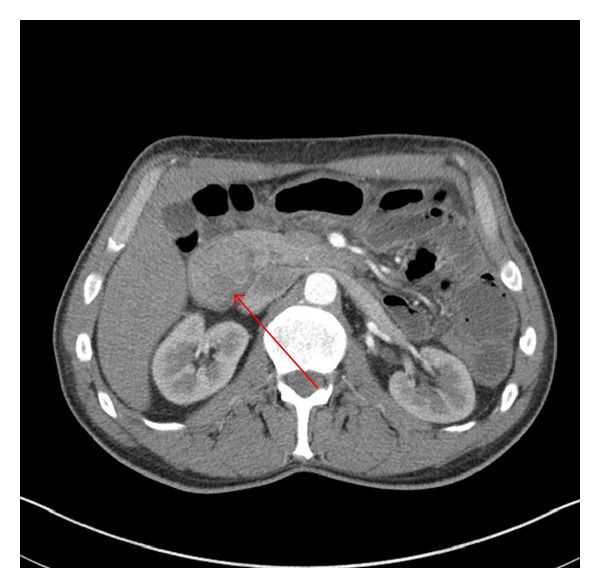
Image from contrast enhanced abdominal CT scan confirming presence of mass lesion in the duodenum (arrow).

An upper GI series barium study demonstrated a large nonmobile filling defect within the second portion of the duodenum worrisome for a large mass (Figure 2). Contrast enhanced CT of his abdomen showed a 4.5 × 3.4 cm mass in the second portion of the duodenum along with additional smaller soft tissue masses in loops of proximal small bowel (Figure 3). Upper and lower endoscopy confirmed an obstructive mass in the duodenum as well as numerous 0.2–0.4 cm polyps in the remaining segment of colon. Pathology of biopsied specimens from both large and small bowel revealed mild acute inflammation superimposed on architectural disorganization without dysplasia, suggestive of Peutz-Jeghers polyps. 

**Figure 4:**
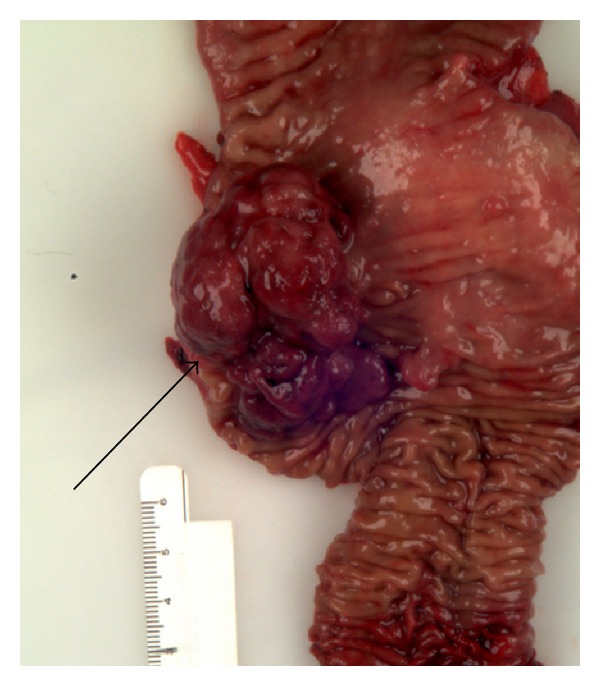
Surgical specimen from duodenal resection with the lumen opened displaying the inner bowel mucosa and mass (arrow).

Due to the severe weight loss and obstructive symptoms, the patient underwent surgical resection (Figure 4). A small bowel resection with primary anastomosis and a classic Whipple procedure were performed due to the extent of disease in the duodenum. 

Following surgery the patient had an uncomplicated recovery in the hospital and was discharged 8 days later. His diet was progressed steadily and he began slowly weaning off pancreatic enzyme supplements. The patient was educated on PJS and placed on an appropriate surveillance regimen.

## 3. Discussion

The diagnostic criteria for PJS include the presence of small bowel hamartomatous polyps, characteristic mucocutaneous pigmentation, and family history [7]. Two of these criteria must be met in order to make a clinical diagnosis of PJS. Endoscopically, the polyps in PJS have no specific features and are grossly indistinct from other hamartomatous polyposis syndromes. Definitive characterization is made with histology. PJS polyps demonstrate smooth muscle proliferation and an elongated arborized pattern of polyp formation [8]. Histopathologic diagnosis will reliably differentiate a hamartomatous polyp from an adenomatous polyp, as can be seen in familial adenomatous polyposis (FAP), or a mixed adenomatous and hyperplastic polyp seen in Hereditary Mixed Polyposis Syndrome. Recognition of characteristic skin lesions combined with thorough family history can direct the clinician towards an appropriate diagnosis of PJS in the absence of definitive histopathology.

A significantly increased risk of both gastrointestinal and nongastrointestinal malignancies has been demonstrated for patients with PJS. Studies have reported PJS patients having a relative risk of cancer ranging from 10 to 18 when compared to the general population [9]. By the time they are 65 years old PJS patients have up to a 93% chance of developing a malignancy [10]. Nongastrointestinal malignancies for which PJS patients are at higher risk include breast, lung, ovarian, cervical, and testicular cancers [6].

Increased relative risk of malignancies for PJS patients has necessitated the implementation of a comprehensive screening and surveillance program as part of clinical management. In 2007, European experts crafted a consensus statement and proposed surveillance guidelines for the management of these patients. Their recommendations included video capsule endoscopy every three years beginning at 8 years of age, colonoscopy and endoscopy every three years from age 18 on (and in some cases earlier), colonoscopy every one to two years after age 50, and annual breast MRI from age 25 to 50 followed by annual mammography, as well as other testings [11].

In this case the patient presented without a confirmed history of PJS. Despite the classic physical exam findings and known intestinal polyposis, an appropriate surveillance program had not been implemented presumably because the definitive diagnosis had not been made. It is unclear why this patient, which was previously found to have a dysplastic polyp and a suspected polyposis syndrome, only underwent a partial colectomy. If a diagnosis of familial adenomatous polyposis had been incorrectly made, then the patient should have underwent a total proctocolectomy. Treatment for colonic polyps in patients without a polyposis syndrome typically depends on the type of polyp, size, and degree of dysplasia. PJS patients are estimated to have a 39% cumulative risk for developing colorectal cancer from age 15 to 64 [10], which has prompted the recommendations for frequent colonoscopic screening. Without proper surveillance, specifically upper endoscopy, this patient developed a large obstructing duodenal mass requiring surgery with wide excision and its own set of substantial comorbidities. Surveillance may have detected the duodenal mass at an earlier stage and a less extensive procedure could have been performed.

## 4. Conclusion

This case demonstrates a classic clinical presentation of a patient with Peutz-Jeghers syndrome. PJS patients have increased risk of malignancy which includes nongastrointestinal cancers and therefore correct diagnosis and appropriate surveillance are important. Proper surveillance for PJS related disease can result in preventable comorbidities. Surgery may be required for obstructive gastrointestinal lesions as well as those exhibiting malignant degeneration.
